# Microbial Community Profiles in Wastewaters from Onsite Wastewater Treatment Systems Technology

**DOI:** 10.1371/journal.pone.0147725

**Published:** 2016-01-25

**Authors:** Łukasz Jałowiecki, Joanna Małgorzata Chojniak, Elmar Dorgeloh, Berta Hegedusova, Helene Ejhed, Jörgen Magnér, Grażyna Anna Płaza

**Affiliations:** 1 Department of Environmental Microbiology, Institute for Ecology of Industrial Areas, Katowice, Poland; 2 Development and Assessment Institute in Waste Water Technology at RWTH Aachen University, Aachen, Germany; 3 Natural resources &Environmental Effects, IVL Swedish Environmental Research Institute, Stockholm, Sweden; NERC Centre for Ecology & Hydrology, UNITED KINGDOM

## Abstract

The aim of the study was to determine the potential of community-level physiological profiles (CLPPs) methodology as an assay for characterization of the metabolic diversity of wastewater samples and to link the metabolic diversity patterns to efficiency of select onsite biological wastewater facilities. Metabolic fingerprints obtained from the selected samples were used to understand functional diversity implied by the carbon substrate shifts. Three different biological facilities of onsite wastewater treatment were evaluated: fixed bed reactor (technology A), trickling filter/biofilter system (technology B), and aerated filter system (the fluidized bed reactor, technology C). High similarities of the microbial community functional structures were found among the samples from the three onsite wastewater treatment plants (WWTPs), as shown by the diversity indices. Principal components analysis (PCA) showed that the diversity and CLPPs of microbial communities depended on the working efficiency of the wastewater treatment technologies. This study provided an overall picture of microbial community functional structures of investigated samples in WWTPs and discerned the linkages between microbial communities and technologies of onsite WWTPs used. The results obtained confirmed that metabolic profiles could be used to monitor treatment processes as valuable biological indicators of onsite wastewater treatment technologies efficiency. This is the first step toward understanding relations of technology types with microbial community patterns in raw and treated wastewaters.

## Introduction

Wastewater treatment approaches vary from the conventional centralized systems to the entirely onsite decentralized and cluster systems. The centralized wastewater collection and treatment systems are costly to build and operate as they treat large volumes of wastewater from large communities requiring the use of large pipes, major excavation and manholes for access. Alternatively, the decentralized approach for wastewater treatment contains a combination of onsite and/or cluster systems for treating wastewater from individual homes and buildings and are designed to operate on a small scale. The decentralized systems collect, treat and reuse/dispose treated wastewater at or near the generation point. They not only reduce the effects on the environment and public health but also increase the ultimate re-use of wastewater using technical options in local settings. Moreover, decentralized systems can be installed on an as needed basis and are particularly more preferable for communities with improper zoning such as low-density populated areas. Massoud *et al*. [1] review the various decentralized approaches to wastewater treatment and management. The small unit size of decentralized systems allows for closer matching to a growing demand and provides a “build-as-you-need” or “just-in-time” capacity [2]. Most onsite wastewater treatment systems are of the conventional type, consisting of a septic tank and a subsurface wastewater infiltration system (SWIS). Site limitations and more stringent performance requirements have led to significant improvements in the design of wastewater treatment systems and how they are managed. Over the past 20 years the onsite wastewater treatment system (OWTS) industry has developed many new treatment technologies that can achieve high performance levels on sites with size, soil, ground water, and landscape limitations that might preclude installing conventional systems. New technologies and improvements to existing technologies are based on defining the performance requirements of the system, characterizing wastewater flow and pollutant loads, evaluating site conditions, defining performance and design boundaries, and selecting a system design that addresses these factors [1].

The most common and traditional of onsite wastewater treatment system (OWTS) is the septic system consisting of a septic tank that has gravity flows to a soil adsorption field. Scientists, engineers, and manufacturers in the wastewater treatment industry have developed a wide range of alternative technologies designed to address increasing hydraulic loads and water contamination by nutrients and pathogens. However, most of the alternative treatment technologies applied today treat wastes after they exit the septic tank. Post-tank treatment can include aerobic or anaerobic biological treatment in suspended or fixed-film reactors, physical/chemical treatment, soil infiltration, fixed-media filtration and/or disinfection. The application and sizing of treatment units based on these technologies are defined by performance requirements, wastewater characteristics, and site conditions [3].

Among the various ecosystems, effluent treatment plants (ETPs) represent microbial communities existing as dynamic consortia. The different co-existing microbial populations in wastewaters change with reactor operational conditions [4,5,6]. Their contribution to overall degradation is likely to provide unprecedented control over the bioremediation of the effluents [7,8]. The microbial diversity of two different effluent treatment plants of pesticide and pharmaceutical industries was characterized using culture-dependent and independent approaches [9]. The authors used distance-based operational taxonomic unit and richness (DOTUR) to calculate diversity indices and richness estimators. In this method sequences were grouped as operational taxonomic units (OTUs) or phylotypes, both of which were defined by DNA sequences.

Whole effluent biological methodologies (eco- toxicological bioassays) are often incorporated in evaluation of water quality from centralized effluent discharges [10–11] but these approaches have not been employed to assess onsite effluent water quality. The quality of onsite effluent discharges is evaluated with basic physic-chemical measures [12]. In the paper written by Garcia *et al*. [13] the effluent water quality from a municipal treatment plant and two onsite wastewater treatment systems was evaluated by tiered testing approach included: routine water quality parameters (Tier I), toxicity using *D*. *magna* bioassay (Tier II), and level of select endocrine-active steroids (Tier III).

Nevertheless, it seems necessary to take under consideration the rapid community-level cultural approach called CLPPs based on Biolog^™^ microtiter plates in determining the functional diversity of microbial communities in the wastewater. The Biolog^™^ system has been extensively used in applied ecological research to identify microbes and to detect changes mainly in soil microbial communities [14–21]

The aim of the study was to determine the potential of CLPPs methodology as an assay for characterization of the metabolic diversity of wastewater samples and to link the metabolic diversity patterns to efficiency of select onsite wastewater facilities. Metabolic fingerprints obtained from the selected samples were used to understand functional diversity implied by the carbon substrate shifts.

## Materials and Methods

### Description of onsite wastewater treatment systems

The facilities are located in the testing field at PIA (Development and Assessment Institute in Waste Water Technology, RWTH Aachen University, Germany). The daily hydraulic flow of the WWTPs is 0.75 m^3^/d. Studied wastewater treatment plants differ in terms of their treatment capacity and the type of treatment technology. Technologies A and B are based on the biofilm technology where microorganisms degrade organic contaminants in the wastewater while being attached to different carrier materials and forming a biofilm. Technology C uses a combination of the activated sludge technology and the biofilm technology. Oxygen needed for the degradation of the organic contaminants in the wastewater is supplied through aeration systems that are also technology dependent. Additional microbiological differences between technologies A, B and C could be also explained by their specific bioreactor characteristics.

**Fig 1:**
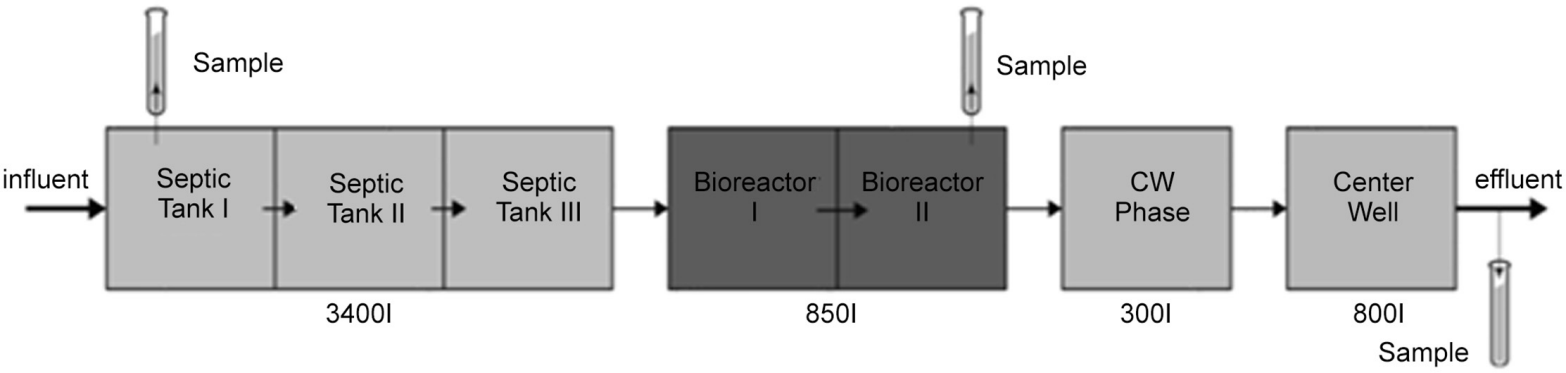
Technology A—Fixed bed reactor.

Technology A- *Fixed bed reactor* (Fig 1) is a treatment integrating mechanical, biological and chemical elements. All purification steps are combined in a single container. The plant is divided into six chambers. Technology A uses a fixed bed reactor where the media stays in one position as the wastewater flows past. Growth support medium is fixed in space by gravity. The biological wastewater treatment is carried out by microorganisms attached to the carrier material installed in the bioreactor and covered with water. Because the biofilm remains attached to a solid surface it is not possible to analyze the filter material colonized with microorganisms. However, there should be enough suspended microorganisms removed from the fixed bed that can be used for the bioreactor sampling.

**Fig 2:**
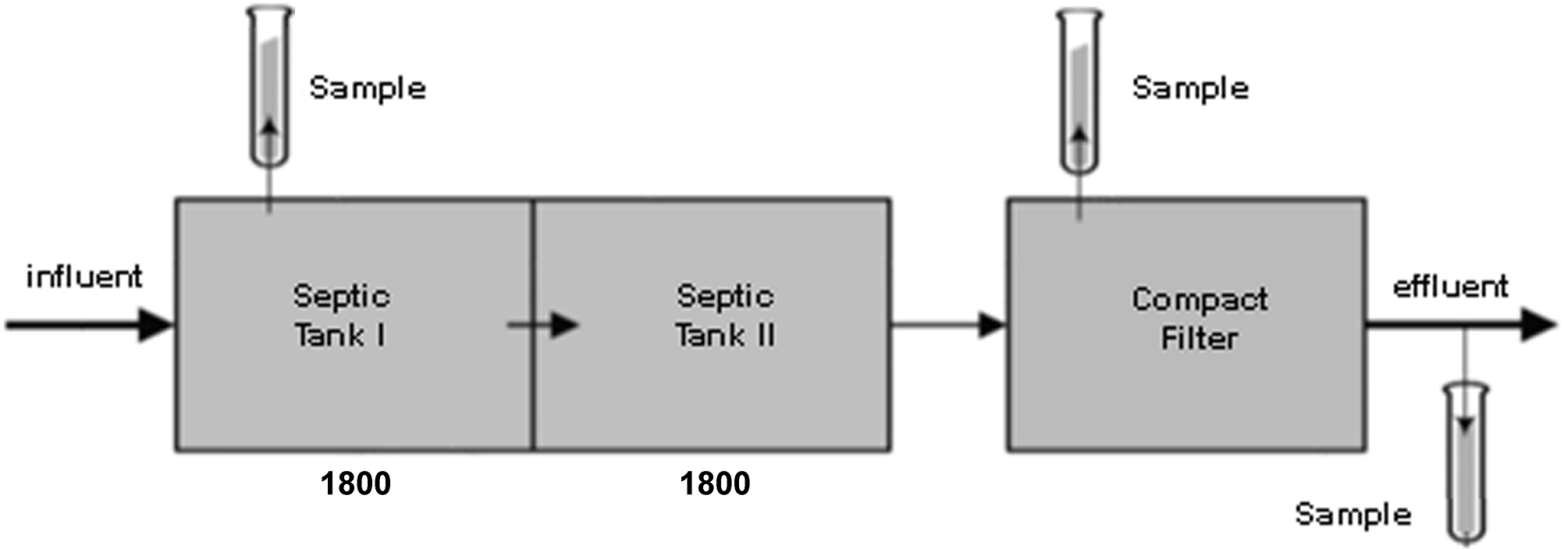
Technology B–Trickling filter/biofilter system.

Technology B*- Trickling filter/biofilter* system (Fig 2) uses a two staged treatment system. The system consists of two septic tanks and a compact filter. The compact filter is made of rock wool which is manufactured into cubes which differ in size. The bioreactor of technology B is called a trickling filter. Trickling filter is a type of biofilter used in wastewater treatment with attached biomass on the filter-media. In this trickling filter consisting of rock wool media wastewater flows over the filter and causes layers of biomass to be formed. This process enables free-floating microbes to create a complex community holding to the surface of the filter media they have settled on. Therefore, the filter filled with rock wool acts as a high surface area for biofilm formation where organisms grow over the surface of the media. This type of reactor retains media in suspension.

**Fig 3:**
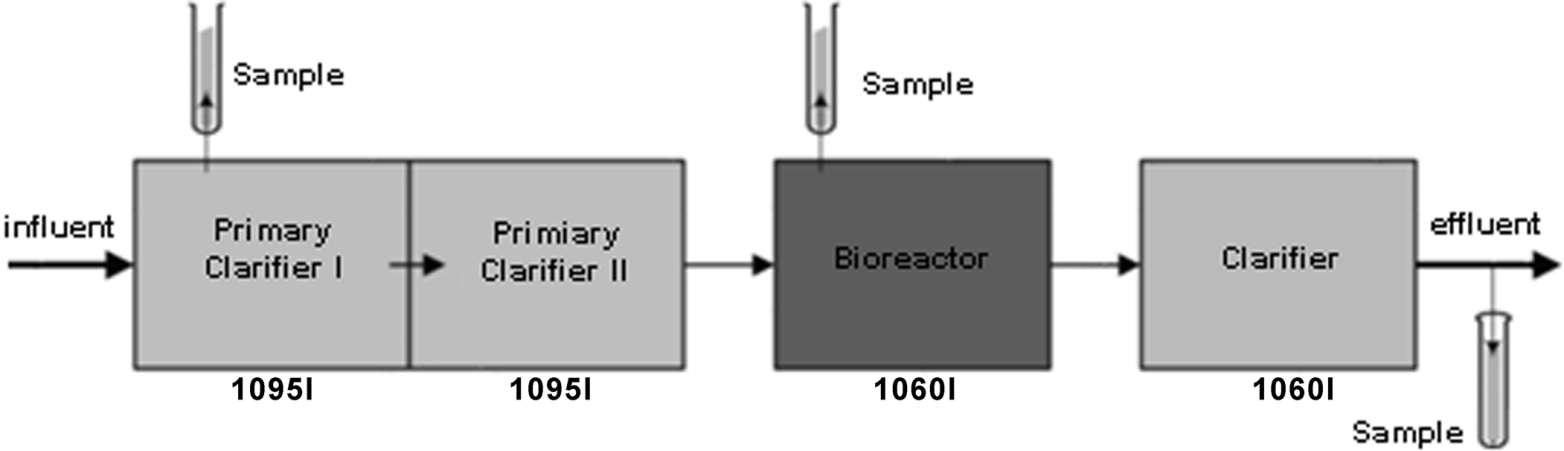
Technology C–Aerated filter system—The fluidized bed reactor.

Technology C *Aerated filter system—The fluidized bed reactor* (Fig 3) operates on a principle of a fluidized bed biological reactor with fluidized media providing a high active surface for microorganisms growing on it. In the third tank which is the bioreactor, the biological treatment takes place during which wastewater is aerated and mixed. The air is being dosed into the reactor to support oxygenation and mixing. The bioreactor functions with continuously moving media. Microorganisms are immobilized on the small, fluidized units of carrier media which make the treatment process to be operated with a minimal biomass wash-out. Wastewater is pumped upward through a bed of media resulting in fluidization of the carrier media. There are also suspended microorganisms in the bioreactor which are released from the fluidized media. Microorganisms which are also sloughed from the surface of carrier media were collected as part of a liquid sample. For the analysis of those microorganisms which create the biofilm growing attached to the carrier media that remain fluidized, a biofilm covered carrier media were taken. It is assumed that the concentration of active biomass should be higher in this technology than in the other two technologies because of the greater available surface area for biological growth.

### Sampling

In order to catch the variability of microbial parameters in influent, effluent and bioreactor from different wastewater treatment plants, raw influent, effluent and bioreactor grab samples were taken. Three samples per each plant were taken at the inlet and outlet, as well as directly from the bioreactors of technologies A, B and C. All three technologies were sampled on the same day. The samples were collected from three wastewater treatment plants (WWTPs) on two occasions– 20.11.2014 and 19.2.2015. The total number of samples was 18. The following samples were collected:

Technology A: influent, effluent, sludge (liquid from the bioreactor)
Technology B: influent, effluent, sludge (rock wool pieces)
Technology C: influent, effluent, sludge (carrier media, liquid from the bioreactor)

All grab (or catch) samples were collected manually by trained personnel. Properly prepared and uncontaminated labeled and dated sampling containers and gear were used. A 1000 ml volume was chosen for every sample. The sample material was placed immediately in a plastic, screw-capped container and the containers were placed in a shipping box. Appropriate sample storage conditions were ensured together with the shortest transport and storage time. The samples were cooled down but not frozen, with recommended temperature kept under 4°C. Holding time was under 24 hours.

### Enumeration of culturable aerobic microorganisms

The number of viable heterotrophic bacteria and fungi were determined by cultivation of microorganisms on selective media. Indirect standard method based on the growth of microorganisms from the samples and plating serial dilutions on a solid agar medium was used. For each sample 10 mL of samples were placed in Erlenmeyer flasks containing 90 mL of 0.85% sterile (filtered) NaCl (pH 7.0–7.2) for shaking (180 rpm, 20 min). Serial tenfold dilutions of samples were surface spread onto selective agar plates. Cycloheximide (100 mg L^-1^) was added to the medium to inhibit fungal growth. SMA (Standard Methods Agar, BioMérieux) was used to determine numbers of viable heterotrophic bacteria. Colonies were enumerated after 72 hours incubation at 22°C. Fungi were incubated on MEA medium (Malt Extract Agar, BioMérieux) with 100 mg L^-1^ chloramphenicol at 22°C for 7 d.

### Community level substrate utilization analysis

Biolog EcoPlates (Biolog, Hayward, CA) are 96-well plates, containing three replicate sets of 31 different substrates, which are ecologically relevant, structurally diverse compounds (Table 1). These substrates are widely used to assess functional diversity of soil microbial communities and are based on community-level carbon sources utilization patterns [19]. Tetrazolium violet redox dye was used for each well as a color indicator if added microorganisms utilize the substrates. 10 ml of samples were shaken in 90 ml of distilled sterile water for 20 min at 25°C. Next 150 μl of each sample were inoculated into each well of Biolog EcoPlates and incubated at 22°C. The rate of utilization was indicated by the reduction of the tetrazolium, a redox indicator dye that changes from colorless into purple. The color development was read as absorbance every 24 h with Microstation (Biolog Inc.) at a wavelength of 590 nm. The data were collected using Microlog Data Collection Software 1.2 (Biolog Inc.).

**Table 1:** Biolog EcoPlates carbon source guild groupings [24].

Well number	Carbon source	Compound group
A1	Water	-
B1	Pyruvic acid methyl ester	Carbohydrates
C1	Tween 40	Polymers
D1	Tween 80	Polymers
E1	α- Cyclodextrin	Polymers
F1	Glycogen	Polymers
G1	D-Cellobiose	Carbohydrates
H1	α-D-Lactose	Carbohydrates
A2	β-Methyl-D-glucoside	Carbohydrates
B2	D-Xylose	Carbohydrates
C2	i-Erythritol	Carbohydrates
D2	D-Mannitol	Carbohydrates
E2	N-Acetyl-D-glucosamine	Carbohydrates
F2	D-Glucosaminic acid	Carboxylic & Acetic acids
G2	Glucose-1-phosphate	Carbohydrates
H2	D,L-α-Glycerol phosphate	Carbohydrates
A3	D-Galactonic acid-γ-lactone	Carboxylic & Acetic acids
B3	D-Galactyronic acid	Carboxylic & Acetic acids
C3	2-Hydroxybenzoic acid	Carboxylic & Acetic acids
D3	4-Hydroxybenzoic acid	Carboxylic & Acetic acids
E3	γ-Hydroxybutyric acid	Carboxylic & Acetic acids
F3	Itactonic acid	Carboxylic & Acetic acids
G3	α-Ketobutyric acid	Carboxylic & Acetic acids
H3	D-Malic acid	Carboxylic & Acetic acids
A4	L-Arginine	Amino acids
B4	L-Asparagine	Amino acids
C4	L-Phenyloalanine	Amino acids
D4	L-Serine	Amino acids
E4	L-Threonine	Amino acids
F4	Glycyl-L-glutamin acid	Amino acids
G4	Phenylethylamine	Amines & Amides
H4	Putrescine	Amines & Amides

Microbial response in each microplate that expressed average well-color development (AWCD) was determined by Gomez *et al*. [22]

$$AWCD=\sum \frac{ODi}{31}$$

where ODi is optical density value from each well, corrected subtracting the blank well (inoculated, but without a carbon source).

AUC (Area Under the Curve) was calculated as:

$$AUC=\sum \frac{A_{n}+A_{n+1}}{2\times (t_{n+1}-t_{n}}$$

where A_n_ and A_n+1_is the absorbance of each individual well at two consecutive measurements at two different measurement times for t_n_ and t_n+1_.

Biolog data incubated for 72 h were analyzed according to Zak *et al*. [23] to give substrate richness (catabolic richness) (S) values, e.g. total number of oxidized C substrates = total number of wells with absorbance over 0.25, and catabolic diversity index (Shannon-Weiner functional diversity index, H). The Shannon-Weiner functional diversity index was calculated as:

H=−∑pi(lnpi)

where pi is the ratio of the activity on each substrate (ODi) to the sum of activities on all substrates ∑ODi.

Shannon Evenness (E) index was calculated from Shannon-Weiner diversity index (H) and substrate richness (S) index as follows:

$$E=\frac{H}{lnS}$$

The five guilds of carbon substrates proposed by Weber and Legge [24] were used:

1) carbohydrates (Carb,), 2) carboxylic and acetic acids (C & AA), 3) amino acids (AA), 4) polymers (Poly), and 5) amines and amides (A & A) (Table 1). The carbon sources, selected as miscellaneous by Zak *et al*. [23], were included into carbohydrates category according to Weber and Legge [24]. For each guild the corrected absorbance values of the substrates were summarized and expressed as a percentage of total absorbance value of the plate [24].

### Statistical analysis

Principal components analysis (PCA) and cluster analysis (nearest neighbor method with Euclidian distance) were used to determine differences between patterns of the substrate utilization profiles (CLPPs) of investigated communities. To compare the utilization profiles, the samples were compared with the 31 variables (C substrates). Metabolic profiles 31 of five carbon substrate groups were analyzed using principal component analysis (PCA). The PCA is a multivariate statistical analysis technique used to project the maximum variance of the average absorbance data of five carbon substrate groups in multiple dimensions (e.g., axis 1 and axis 2), in an unconstrained ordination. In the PCA ordination diagram, treatments with similar patterns of relative absorbance of substrate groups are located close to one another, while treatments with different patterns are far apart. Principle component analysis (PCA) to analyze CLPPs was performed on normalized and transformed absorbance data for each well, according to Weber *et al*. [25]. All statistical analyses were performed with Statistica 10.0 software (2011).

## Results and Discussion

**Fig 4:**
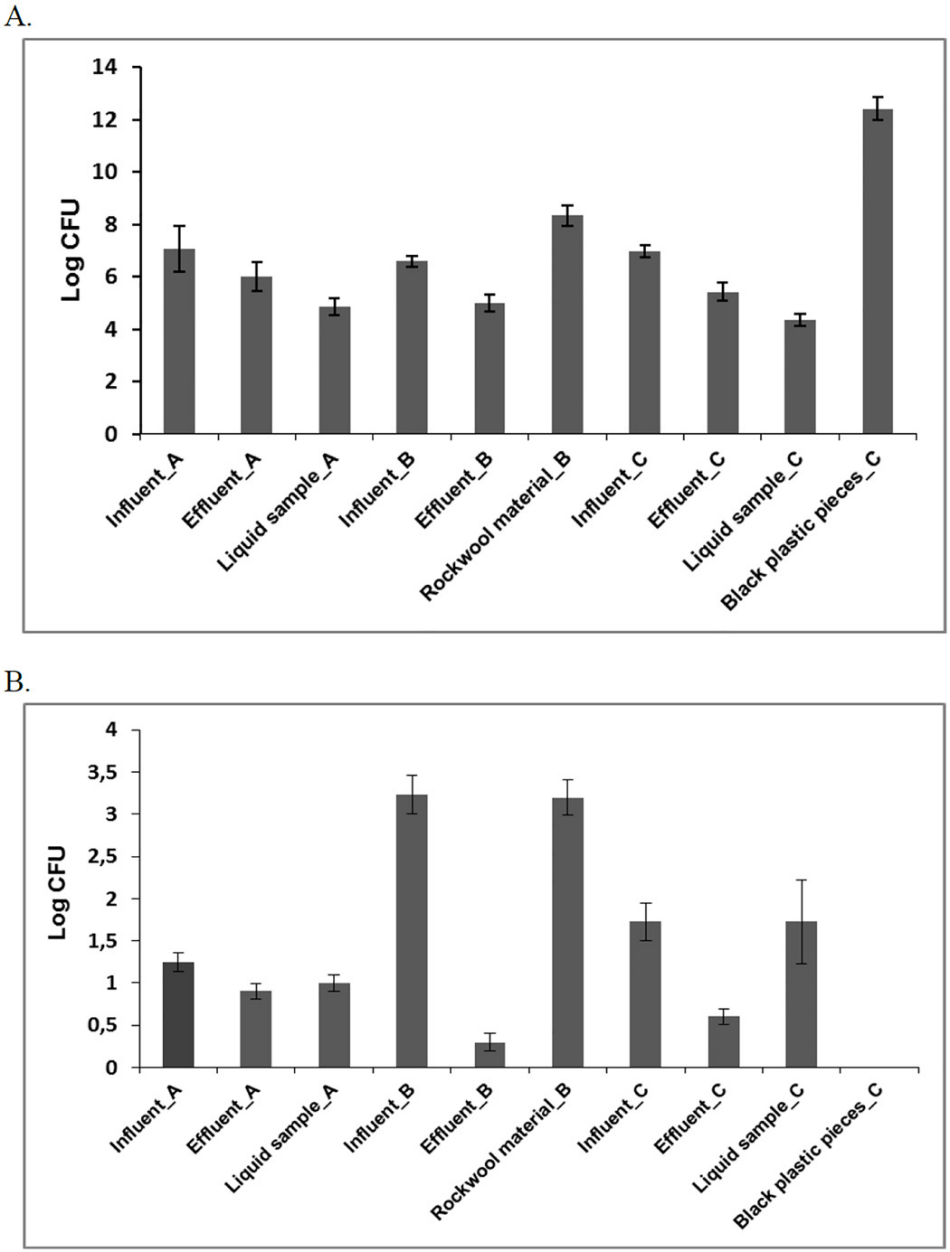
Average number of bacteria (A) and fungi (B) in raw and treated wastewaters. Bars represent the standard deviation.

Some physico-chemical parameters and number of microorganisms in raw and treated wastewaters are presented in Table 2 and Fig 4, respectively. Technologies B and C had the similar effictiveness in reduction of microorganisms number. The reduction of number of bacteria and fungi in effluents from technology B and C was 25% and 90%, and 22% and 65%, respectively. The capacity of bacterial communities to utilize a set of sole (Biolog EcoPlates^™^ assay) carbon sources was tested in order to evaluate the microbial efficiency of the selected technologies. In Table 3 the values of biodiversity indices for the onsite wastewater technologies are presented. The Shannon index (H) separated the bacterial physiological diversity referring to the technologies applied. The highest values of H were noted for the rockwool material (technology B) and black plastic pieces (technology C). These materials were used as sorbent of sludge and microorganisms. The rate of Shannon index is affected by both substrate richness (number of positive wells) and substrate evenness (equality of wells’ optical densities). In this case, similar relationships were observed for these indices. The similar values were observed for AWCD and AUC in the samples. According to the obtained indices, the most active on EcoPlates^™^ were the heterotrophic bacteria from the rockwool material (technology B) and black plastic pieces (technology C). The results obtained indicated higher values of AWCD, H, E, AUC values in influents compared to the effluents for selected technologies. The reduction in functional diversity was significantly greater in effluents of the investigated technologies. These differences were probably due to the presence of more organic compounds in effluents, biodegradation potential of microbial communities, microbes density, and effectivity of applied technologies.

**Table 2:** Basic parameters of raw and treated wastewaters (mean values).

No.	Characteristics	Units	Influent			Effluent		
			Technology			Technology		
			A	B	C	A	B	C
**1.**	Temperature	[°C]	10.13±0.07			9.15	9.45	8.8
**2.**	COD	[mg/l]	730.5±1.41			nd	nd	nd
**3.**	NH_4_-N	[mg/l]	32.08±0.28			nd	nd	nd
**4.**	N_tot_	[mg/l]	60.83±1.41			nd	nd	nd
**5.**	P_tot_	[mg/l]	7.18±0.42			nd	nd	nd
**6.**	pH	[–]	7.46±0.02			8.95	7.59	7.53
**7.**	Conductivity	[uS/cm]	838.83±2.82			765	713.5	691
**8.**	Turbidity	[FNU]	175.5±9.19			6.7	10.7	7.6
**9.**	Settleable solids	[ml/l]	nd			<0.1	<0.1	0.3

nd–no detected.

**Table 3:** The biodiversity indices values of the collected samples from the facilities.

Samples	Indices				
	AWCD	H	E	S	AUC
**TECHNOLOGY A**					
**Influent**	0.869±0.019	1.403±0.011	1.004±0.007	25±0.00	541.36
**Effluent**	0.019±0.006	1.183±0.058	0.00	0.00	7.02
**Liquid sample (bioreactor)**	0.504±0.004	1.342±0.005	1.115±0.025	16±1.00	168.47
**TECHNOLOGY B**					
**Influent**	0.994±0.028	1.423±0.005	0.991±0.012	27±1.16	621.41
**Effluent**	0.178±0.068	1.467±0.011	0.988±0.007	0.00	225.00
**Rockwool material**	1.302±0.045	1.482±0.003	0.993±0.002	31±0.00	857.00
**TECHNOLOGY C**					
**Influent**	0.663±0.022	1.406±0.005	0.942±0.004	24.00	320.79
**Effluent**	0.794±0.015	1.412±0.005	0.947±0.003	24±0.58	283.34
**Black plastic pieces**	1.230±0.039	1.469±0.004	0.985±0.003	31.00	778.93
**Liquid sample (bioreactor)**	0.533±0.017	1.369±0.007	0.918±0.005	21±1.16	232.37

Mean values ± Stand. Dev.

AWCD—average well-color development; H index–Shannon-Weiner functional diversity index; E–Shannon Evenness index; S–catabolic richness; AUC–area under the curve.

The Shannon index separates the physiological diversity of microbes according to the technology specificity. The substrate richness and substrate evenness have different weight in the value of bacterial physiological diversity [17]. The results obtained by Wittebolle *et al*. [26] present that the bacterial community evenness is more indicative in polluted sites. The biodiversity is a good indicator of rapid response of natural community to selective stress and thereby plays a role for ecosystem functionality.

**Fig 5:**
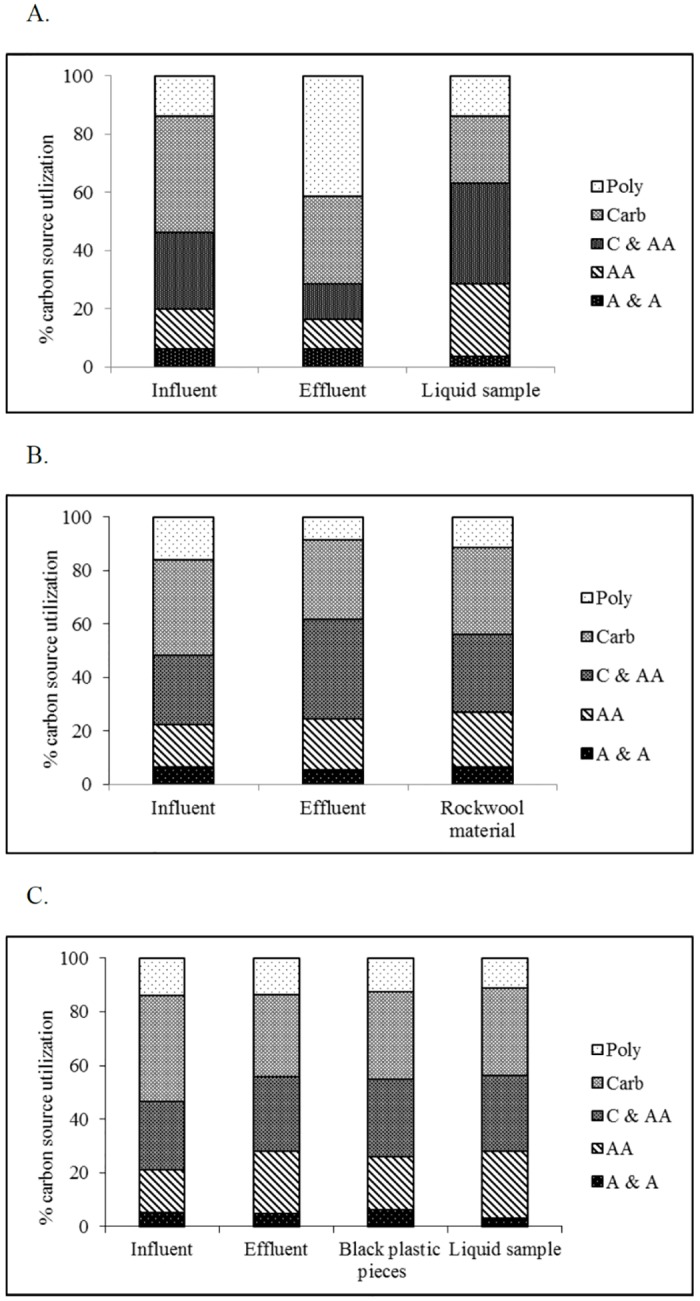
Percent of carbon source utilization response for the investigated samples. A–the samples from technology A; B–the samples from technology B; C- the samples from technology C. The carbon sources were divided in the following guilds: amines and amides (A & A), aminoacids (A), carboxylic and acetic acids (C & AA), polymers (Poly) and carbohydrates (Carb).

**Fig 6:**
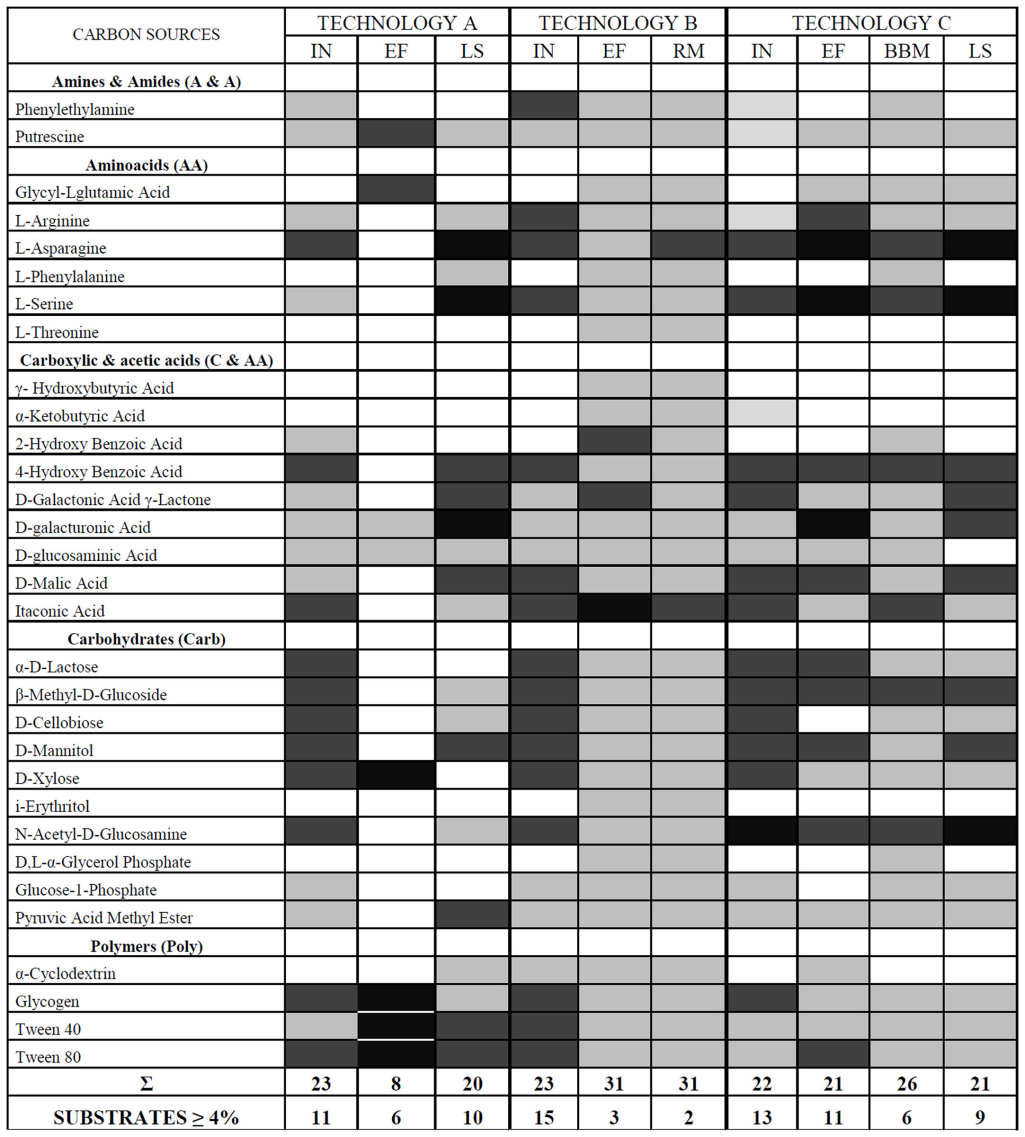
Pattern of utilization (based on mean AWCD) of the 31 carbon sources for the investigated samples from the biological onsite wastewater technologies. Shading in the boxes indicates the range of percentage absorbance of the total absorbance of the plate. Values are as follows: white < 2%; light grey 2–4%; dark grey 4–6%; black > 6%. Abbreviations: **IN**- influent; **EF**- effluent; **LS**- liquid samples from the bioreactor; **RM**- rockwool material; **BBM**- black plastic pieces. Below each column, the number of substrates with greater or equal to 4% absorbance for each sample is indicated.

Functional diversity, understood in this study as the utilization of carbon sources in Biolog EcoPlates, showed distinct differences between the samples and technologies (Fig 5). In general, investigated samples were characterized by the different utilization pattern of the five guilds. Especially the big differences were noted between the samples from technology A. However, a similar pattern of carbon substrate utilization was shown in the samples from technologies B and C. The insignificant differences among the AWCDs manifest the equivalent potential of microbes to utilize a set of natural relevant carbon sources. The high catabolic capacity of rockwool material and black plastic pieces may refer to a high number of heterotrophic bacteria and changed community composition. Heterotrophic bacteria utilized more intensively carboxylic and acetic acids and carbohydrates in all samples of investigated technologies. Most of the carboxylic and acetic acids and carbohydrates are intermediates of organic matter degradation [27]. The influents from the investigated technologies were characterized by higher utilization of the substrates. Similar pattern of carbon utilization was presented in all analyzed influents. However, the differences were observed in the effluents from the investigated technologies. The similar pattern of carbon utilization was only obtained in effluents for the two technologies: B and C. The results suggested that microbial efficiency of B and C technologies was similar. The number of utilization substrates in influents (> 4%) was 11, 15, 13 of the 31 available carbon substrates in the technologies A, B and C, respectively (Fig 6). Interestingly, the number of utilized substrates for rockwool material and black plastic pieces was higher, 31 and 26, respectively, but the activity level to utilize the substrates was lower compared to the other samples. Only L-asparagine and itaconic acid were utilized with activity above 4% for rockwool material (technology B). Six substrates (L-asparagine, L-serine, 4-hydroxy benzoic acid, itaconic acid, β-methyl-D-glucoside, N-acetyl-D-glucosamine) were utilized with the similar level of activity for black plastic pieces (technology C) (Fig 6). On the other hand, the number of utilization of 31 carbon sources in the effluents from technologies A and B was lower 6 and 3, respectively.

**Fig 7:**
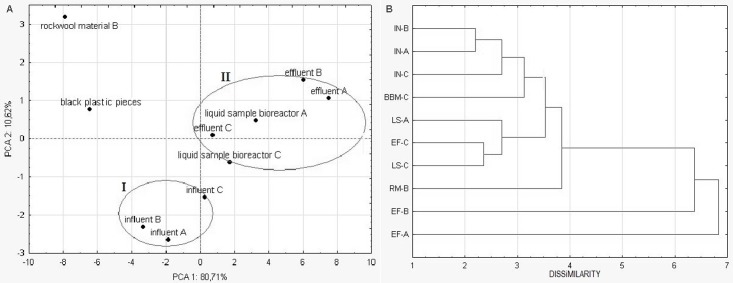
A—Dendrogram of the metabolic fingerprints of tested microbial communities by cluster analysis of grouped Eco data set. B—Bidimensional plot of principal component analysis for Biolog Eco profiles of microbial communities. PC1 and PC2 refer to the first two principal components, accounting for 81% and 11% of total variance, respectively. Abbreviations: **IN**- influent; **EF**- effluent; **LS**- liquid samples from the bioreactor; **RM**- rockwool material; **BBM**- black plastic pieces.

The results of statistical analysis are presented in Fig 7A and 7B. The bidimensional plot (PC1 x PC2) presented in Fig 7A shows the relationships among 10 microbial communities according to EcoPlates carbon-source utilization pattern, where PC1 accounted for 81% of the total variation observed and PC2 explained 11%. Principal component analysis was done based on adjusted average well color development. The neighboring microbial communities in the scatterplot were expected to have similar carbon source use, whereas samples with a large distance to each other were expected to be different according to carbon source use. In this context, according to PC, which is the axis explaining the greater variance of the original data (85%) and has most of the substrates associated with it, the microbial communities were grouped into two major clusters named I and II according to similarity of carbon source use, relative to the 31 substrates. The cluster I contains the communities of influents A, B and C. While, the communities for effluents A, B and C, and liquid samples A and B belong to the cluster II. The communities of rockwool material B and black plastic pieces C were observed to be the most different from the communities of cluster I and II. PCA for Eco data showed that influent CLPPs were more similar between the samples. Similar results were obtained for the effluent CLPPs. The dendrogram, obtained by cluster analysis presents alike pattern as obtained from PCA analysis (Fig 7B). Statistical analysis confirmed the results obtained from CLPPs. The results obtained indicated that differences in the composition of influent wastewater and in the plant operation model influenced on activities of microbial communities evaluated by Biolog EcoPlates.

Community-level physiological profiles have been used to differentiate microbial communities in large scale wastewater treatment systems [28–30], in wastewater wetland systems [31], and river water and groundwater. Previous microbiological studies of wastewater systems have been performed by using Biolog GN microplates. Only, Tiquia [32] evaluated the metabolic diversity of water samples on temporal and spatial scales and to link the metabolic diversity patterns to river pollution by using Biolog EcoPlates. Relatively little is known about the changes in diversity of heterotrophic microbial community affect treatment of wastewater, but there is no information on changes of biodiversity in effluents from small wastewater treatment facilities. In this paper, Biolog EcoPlates were used to evaluate the efficiency of the different operation modes on the community metabolic profiles in wastewaters. This is the first step toward understanding relations of biological treatment processes with microbial community patterns. Microplates assay may yield a great deal of information about an important functional attribute of microbial communities and has been shown in some cases to be as sensitive as or more sensitive than other microbial activity parameters [33, 34]. This approach has been proven useful for pinpointing differences in microbial activity and community metabolic profile among and within wastewater-treatment environments [31,34,35].

## Conclusions

Biolog EcoPlates are useful in differentiating between microbial communities, in the determining factors that most influence the separation of these communities and in identifying which substrates were most utilized by the communities. CLPP successfully discriminated among the microbial communities present in raw and treated wastewaters from different technologies. This information is crucial to understanding the physiology of microbial communities in the various environments. The Biolog EcoPlates show a strong potential as an effective ecological indicator of changes in ecosystems.

## References

[1] Massoud MA, Tarhini A, Nasr JA. Decentralized approaches to wastewater treatment and management: Applicability in developing countries. J Environ Managem. 2009;90:652–9. 10.1016/j.jenvman.2008.07.001 18701206

[2] Wang S. Values of decentralized systems that avoid investments in idle capacity within the wastewater sector: A theoretical justification. J Environ Managm. 2014;136:68–75. 10.1016/j.jenvman.2014.01.038 24565878

[3] US Environmental Protection Agency. Handbook for managing on-site and clustered (decentralized)wastewater treatment systems: An introduction to management tools anf information for implementing EPA’s management guidelines. EPA no 832-B-05-001. Office of Water, 2005;Washington, USA.

[4] Kapley A, De Baere T, Purohit HJ. Bacterial diversiy of acivated biomass from a common effluent treatment plant. Res Microbiol. 2007a;158:494–500. 17566710 10.1016/j.resmic.2007.04.004

[5] Kapley A, Prasad S, Purohit HJ. Changes in microbial diversity in fed-batch reactor operation with wastewater containing nitroaromatic residues. Biores Technol. 2007b;98: 2479–84. 10.1016/j.biortech.2006.09.012 17070036

[6] Liu Y, Zhang T, Fang HHP. Microbial community analysis and performance of phosphate removing activated sludge. Biores Technol. 2005;96:1205–14. 10.1016/j.biortech.2004.11.003 15734306

[7] Manefield M, Griffiths RI, Leigh MB, Fisher R, Andrew SW. Functional and compositional comparison oft wo activated sludge communities remediating coking effluent. Environ Microbiol. 2005;7:715–22. 15819853 10.1111/j.1462-2920.2004.00746.x

[8] Paul D, Pandey G, Meier C, van der Meer JR, Jain RK. Bacterial community structure of a pesticide-contaminated site and assessment of changes induced in community structure during bioremediation. FEMS Microbiol. Ecol. 2006;57:116–27. 16819955 10.1111/j.1574-6941.2006.00103.x

[9] Rani A, Porwal S, Sharma R, Kapley A, Purohit HJ, Kalia VC. Assessment of microbial diversity in effluent treatment plants by culture dependent and culture independent approaches. Biores Technol. 2008;99:7098–107. 10.1016/j.biortech.2008.01.003 18280146

[10] US Environmental Protection Agency. Methods for measuring the acute toxicity of effluents and receiving waters to freshwater and marine organisms. EPA-821-R-02-012. Office of Water, 2002; Washington, DC, USA.

[11] US Environmental Protection Agency. On-site wastewater treatment systems manual. EPA/652/R-00/008. Office of Water, Office of Research and Development, 2002; Washington DC, USA.

[12] Nakajima J, Fujimura Y, Inamori Y. Performance evaluation of on-site treatment facilities for wastewater from households, hotels and restaurants. Wat Sci Tech. 1999;39:85–92.

[13] Garcia SN, Clubbs RL, Stanley JK, Scheffe B, Yelderman JC Jr, Brooks BW. (2013) Comparative analysis of effluent water quality from a municipal treatment plant and two on-site wastewater treatment systems. Chemosphere 2013; 92:38–44. 10.1016/j.chemosphere.2013.03.007 23557723

[14] Bochner BR, Gadzinski P, Panomitros E. Phenotype MicroArrays for high-throughput phenotypic testing and assay of gene function. Genome Res. 2001;11:1246–55. 11435407 10.1101/gr.186501 PMC311101

[15] Bradley RL, Shipley B, Beaulieu C. Refining numerical approaches for analyzing soil microbial community catabolic profiles based on carbon source utilization patterns. Soil Biol Biochem. 2006;38: 629–32.

[16] Garland J, Mills A. (1991) Classification and characterization of heterotrophic microbial communities on the basis of patterns of community-level sole-carbon-source utilization. Appl Environ Microbiol. 1991; 57:2351–59. 16348543 10.1128/aem.57.8.2351-2359.1991 PMC183575

[17] Kenarova A, Radeva G, Traykov I, Boteva S. Community level physiological profiles of bacterial communities inhabiting uranium mining impacted sites. Ecotox Environ Safety 2014;100: 226–32. 10.1016/j.ecoenv.2013.11.012 24315773

[18] Kirk JL, Beaudette LA, Hart M, Moutoglis P, Klironomos JN, Lee H, et. al Methods of studying soil microbial diversity. J Microbiol Methods 2004; 58:169–88. 15234515 10.1016/j.mimet.2004.04.006

[19] Preston-Mafham J, Boddy L, Randerson PF. Analysis of microbial community functional diversity using sole-carbon-source utilization profiles–a critique. FEMS Microbiol Ecol. 2002;42:1–14. 10.1111/j.1574-6941.2002.tb00990.x 19709261

[20] Stefanis C, Alexopoulos A, Voidarou C, Vavias S, Bezirtzoglou E. Principle methods for isolation and identification of soil microbial communities. Folia Microbiol. 2013;58:61–8. 22791233 10.1007/s12223-012-0179-5

[21] Widmer F, Flieβbach A, Laczko E, Schulze-Aurich J, Zeyer J. Assessing soil biological characteristics: a comparison of bulk soil community DNA-PLFA-, and Biolog^™^-analyses. Soil Biol Biochem. 2001;33:1029–36.

[22] Gomez E, Garland J, Conti M. Reproducibility in the response of soil bacterial community-level physiological profiles from a land use intensification gradient. Appl Soil Ecol. 2006;26: 21–30.

[23] Zak JC, Willig MR, Moorhead DL, Wildman HG. Functional diversity of microbial communities: A quantitative approach. Soil Biol Biochem. 1994;26:1101–8.

[24] Weber K, Legge R. One-dimensional metric for tracking bacterial community divergence using sole carbon source utilization patterns. J Microbiol Methods 2009;79: 55–61. 10.1016/j.mimet.2009.07.020 19647767

[25] Weber P, Grove J, Gehder M, Anderson W, Legge R. Data transformations in the analysis of community-level substrate utilization data from microplates. J Microbiol Methods, 2007;69:461–9. 17399829 10.1016/j.mimet.2007.02.013

[26] Wittebolle L, Marzorati M, Clement L, Balloi A, Daffonchio D, Heylen K, et.al Initial community evenness favors functionality under selective stress. Nature 2009;458:623–6. 10.1038/nature07840 19270679

[27] Li J, Yu S. Changes in the structure and diversity of bacterial communities during the process of adaptation to organic wastewater. Can J Microbiol. 2010;56:352–5. 10.1139/w10-009 20453903

[28] Cicek N, Macomber J, Davel J, Suldan T, Audic J, Genestet P. (2001) Effects of solids retention time on the performance and biological characteristics of a membrane bioreactor. Water Sci Technol. 2001;43:43–50. 11443985

[29] Van Heerden J, Ehlers M, Cloete T. Biolog for the determination of microbial diversity in activated sludge systems. Water Sci. Technol. 2001;43:83–90. 11379116

[30] Van Heerden J, Korf C, Ehlers M, Cloete T. Biolog for the determination of diversity in microbial communities. Water SA. 2002;28:29–35.

[31] Hench KR, Sexstone AJ, Bissonnette GK. (2004) Heterotrophic community-level physiological profiles of domestic wastewater following treatment by small constructed subsurface flow wetlands. Water Environ Res. 2004;76: 468–73. 15523793 10.2175/106143004x151554

[32] Tiquia SM. Metabolic diversity of the heterotrophic microorganisms and potential link to pollution of the Rouge River. Environ Pollut. 2010;158:1435–43. 10.1016/j.envpol.2009.12.035 20106574

[33] Zhang CB, Wang J, Liu WL, Zhu SX, Ge HL, Chang SX, et.al (2010) Effects of plant diversity on microbial biomass and community metabolic profiles in a full-scale constructed wetland. Ecol Eng. 2010;36:62–8.

[34] Osem Y, Chen Y, Levinsonc D, Hadar Y. The effects of plant root on microbial community structure in aerated wastewater-treatment reactors. Ecol Eng. 2007;29:133–42.

[35] Victorio L, Gilbride KA, Allen DG, Liss SN. (1996) Phenotypic fingerprinting of microbial communities in wastewater treatment systems. Water Res. 1996;30:1077–86.

